# Motherhood a Lost Art?

**Published:** 1920-04-03

**Authors:** 


### Motherhood a Lost Art?

ENGLAND has lost the art of producing mothers, says Dr. J. J. Johnstone, Medical Officer of Health for Leeds. That statement contains something of the exaggeration of the enthusiast, but it is not without elements of truth, as Dr. Johnstone showed by his figures, though only comparative figures can give the true position. A better way of putting it, though not so striking, might have been that England has not yet properly learned the art of motherhood. The total deaths under one year are still very large, and in the majority of cases are due to bad mothering or no mothering at all. A mother's primary duty is to nurse her baby, yet this is a duty—says Dr. Johnstone—that less than 50 per cent. of English mothers perform; hence the high infant death-rate. No mother has any right to delegate the feeding of her baby to a nurse or to a firm of patent-food manufacturers. Ninety per cent. of the mothers could nurse their babies, but the majority do not try. A considerable number, however, try but do not persevere, and it is sometimes the case that the maternity nurses display the same lack of perseverance. Those who refrain from nursing their babies through mere selfishness are undeserving of the high privileges of motherhood; in any case they probably do not value motherhood as a privilege, but regard it as a thing to be taken with the least possible interference with normal modes of life and pleasure.

